# The Assessment of Selected miRNA Profile in Familial Mediterranean Fever

**DOI:** 10.1155/2021/6495700

**Published:** 2021-10-13

**Authors:** Cigdem Yuce Kahraman, Mehmet Ertugrul Egin, Abdulgani Tatar, Hasan Turkez, Adil Mardinoglu

**Affiliations:** ^1^Department of Medical Genetics, Faculty of Medicine, Atatürk University, Erzurum, Turkey; ^2^Department of Medical Biology, Faculty of Medicine, Atatürk University, Erzurum, Turkey; ^3^Centre for Host-Microbiome Interactions, Faculty of Dentistry, Oral & Craniofacial Sciences, King's College London, London SE1 9RT, UK; ^4^Science for Life Laboratory, KTH-Royal Institute of Technology, Stockholm SE-17121, Sweden

## Abstract

Familial Mediterranean fever (FMF) is the most prevalent autoinflammatory disease. Typical findings are recurrent fever attacks with serositis, skin rash, and synovitis. FMF is caused by mutations in the *MEFV* gene, encoding pyrin protein. Pyrin functions in innate immunity and triggers inflammation via inflammatory mediators' production and acts as the primary regulatory component of the inflammasome. On the other hand, various miRNAs play crucial roles in the pathogenesis of different types of cancers and immune-related and neurodegenerative diseases. However, their association with FMF is still unclear. Therefore, in this study, we assessed the roles of selected thirteen miRNAs associated with immune functions. We recruited genetically diagnosed 28 FMF patients and 28 healthy individuals. The expression profiling of the miRNAs was determined by qRT-PCR and normalized to *SNORD61*. Our analysis revealed that miR-34a-5p, miR-142-3p, miR-216a-5p, miR-340-5p, miR-429, and miR-582-5p were upregulated, whereas miR-107, miR-569, and miR-1304-5p were downregulated in the FMF patients. Among them, miR-107 was found to be the most remarkable in M694V homozygous mutants compared to other homozygous mutants. During clinical follow-up of the patients with M694V mutation, which is closely related to amyloidosis, evaluation of mir-107 expression might be crucial and suggestive. Our results showed that miRNAs might serve a function in the pathogenesis of FMF. Further studies may provide novel and effective diagnostic and therapeutic agents that target examined miRNAs. Targeting miRNAs in FMF seems to be promising and may yield a new generation of rational therapeutics and diagnostic or monitoring tools enabling FMF treatment.

## 1. Introduction

Familial Mediterranean fever (FMF) is considered one of the most prevalent autoinflammatory diseases. FMF is more common in individuals of eastern Mediterranean origin. Typical findings are recurrent fever attacks with serositis, skin rash, and synovitis. The diagnosis of FMF is clinically made, particularly in endemic countries. However, it may be difficult to diagnose in some patients with atypical phenotype, and in this case, genetic diagnosis becomes important [1]. FMF is caused by mutations in the *MEFV* gene, encoding pyrin protein, on chromosome 16p13 [2, 3]. Initially, common mutations of the *MEFV* gene are screened, but whole gene sequencing may be requested if necessary. It is known that inflammation markers increase in the blood during attacks. Frequently screened inflammatory markers are leukocyte count, erythrocyte sedimentation rate (ESR), C-reactive protein (CRP), serum amyloid A protein (SAA), and fibrinogen. The most serious complication of FMF is amyloidosis. Amyloidosis refers to the accumulation of amyloid as a result of excessive inflammation in the kidney. Particularly M694V and M694I mutations are associated with severe disease and amyloidosis. Colchicine has been used for centuries to treat FMF and particularly to prevent the amyloidosis. Interleukin-1 (IL-1) antagonists can be tried in cases of resistance or intolerance to treatment [1, 4].

Pyrin has a critical role in innate immunity and triggers inflammation via inflammatory mediators' production and acts as the inflammasome's primary regulatory component. The gain-of-function mutations in the *MEFV* gene lead to excess pyrin production and inflammatory process of FMF [5, 6]. Autosomal recessive form (# 249100) and in recent years, with existing heterozygote *MEFV* mutant families, autosomal dominant form (# 134610) of FMF were defined in the OMIM database. The patients' symptoms are variable, and genotype-phenotype studies revealed M694I and M694V genotypes led to severe phenotype and amyloidosis. The genotype alone might not enable phenotypic differences in patients. It is thought that epigenetic mechanisms, modifier genes, and epigenetic factors may contribute to this variability [4, 5].

miRNAs are associated with several epigenetic mechanisms. They are small, noncoding RNAs that consist of about 22 nucleotides and act in posttranscriptional regulation of gene expression. miRNAs have complementary sequences to the target mRNA, bind the sequence, and regulate the protein expression [7]. miRNAs have variegated regulatory functions in biological processes such as metabolism, cell cycle, stem cell differentiation, viral replication, apoptosis, and immune response [4, 8]. Abnormal miRNA expressions affect these processes and ultimately lead to various pathological consequences. Previous studies revealed that the patterns of miRNA expression were specific to cancer types and immune-related disorders as well as neurodegenerative diseases [9]. Disruption of the miRNA regulatory system in transgenic mice has been reported to cause severe impairment in the mouse immune system and death in the early embryonic period. miRNAs affect cell maturation, proliferation, differentiation, and antibody production and release of inflammatory mediators in the immune system. miRNAs also have essential roles in innate and acquired immune processes [10]. Toll-like receptors (TLRs) are crucial elements of innate immunity and, when activated, lead to the transcription of genes associated with the immune and inflammatory response. Signaling with the lipopolysaccharide receptor, TLR4, differentiated various miRNAs [11]. As described above, miRNAs have effects on immune response as well as in many cellular processes. Therefore, it is thought that miRNAs may also contribute to the pathogenesis of FMF, and there are limited studies in this area [4, 5].

Clinical trials investigate new therapeutic agents that target the epigenetic mechanisms for various diseases, particularly cancers and rare diseases. One of the promising candidates is miRNA-targeted therapies. miRNAs have the potential to be both biomarkers for diagnosis and target molecules for the development of efficient treatment strategies [5]. Cell-free miRNAs in serum or plasma are emerging as stable and reproducible biomarkers, particularly for the diagnosis of cancers and inflammatory-related disorders. Synthetic anti-miRNA oligonucleotides to antagonize target miRNAs or exogenous miRNA mimics that mimic miRNAs' effects are options that might be developed for therapy [12]. Clinical trials investigate new therapeutic agents that target the epigenetic mechanisms for various diseases, particularly cancers and rare diseases. One of the promising candidates is miRNA-targeted therapies. miRNAs have the potential to be targeted therapy molecules in the future, and a number of studies in this field are increasing [13]. Up to date, various studies were performed to reveal the crucial roles of several miRNAs in the pathogenesis of different type of cancers, immune-related diseases, and neurodegenerative diseases [14–16]. However, their association with FMF is still unclear. Therefore, in this study, we aimed to assess the roles of selected thirteen miRNAs associated with immune functions in FMF patients and healthy subjects. We also aimed to perform one of the frontier investigations that will shed light on future miRNA-targeted FMF diagnostic and therapy strategies and contribute to elucidating the pathogenesis of FMF.

## 2. Materials and Methods

### 2.1. Study Groups

The blood samples (2 ml) obtained from the patients were centrifuged at 3000 g. The upper plasma (1 *μ*l) was taken and transferred to Eppendorf tubes. Plasma samples was collected by centrifugation and stored at -80°C for future use. Blood samples were obtained from patients who applied to our clinic with a prediagnosis of FMF between January and March 2020 and had routine mutation analysis. There were 18 females and 10 males in the patient group. 16 females (57.14%) and 12 males (42.86%) were in the control group. The mean age in the control group was 19.96 ± 11.98. Typical clinical findings were abdominal pain, arthritis/arthralgia, and abdominal pain (Table 1).

Among the patients whose genotypes were determined according to FMF mutation analysis results, 17 samples from M694V and other homozygotes and 11 samples from compound heterozygotes were selected (Table 2). The patients were newly diagnosed patients who had not started colchicine yet. 28 healthy individuals who were determined to have no FMF mutation were selected as the control group. The healthy controls recruited were free of cerebrovascular disease, cardiovascular disease, metabolic disease, pregnancy, or lactation. The Atatürk University Ethics Committee of Faculty of Medicine approved the study protocol (Approval No. B.30.2.ATA.0.01.00/61). Parents of the children and adult individuals were informed about the procedure, and written informed consents were obtained from the participants. Our study complied with the Helsinki Declaration principles.

### 2.2. miRNA Expression Study

miScript RNeasy Mini Kit (Hilden, Germany) was used to isolate miRNAs from FMF patient's plasma samples according to the manufacturer's instructions. miRNA quality was measured by MaestroNano Spectrophotometer (Maestrogen, USA). cDNAs were synthesized with Qiagen miScript Reverse Transcription (RT) Kit II (Hilden, Germany), and then cDNA amplification was performed by Qiagen miScript PreAMP PCR kit (Qiagen GmbH, Hilden, Germany). Qiagen miScript Primer Assay kit for miR-103a-3p, miR-107, miR-569, miR-340-5p, miR-34a-5p, miR-582-5p, miR-23a-3p, miR-216a-5p, miR-429, miR-142-3p, miR-449a, miR-34a-5p, and miR-1304-5p primers and miScript SYBR Green PCR kit (Qiagen) were used for miRNA expression study in qRT-PCR (Rotor-Gene Q RT-PCR; Qiagen). RT-qPCR program with 25 *μ*l volume was 40 cycles of 95°C for 2 min, 94°C for 15 s, 55°C for 30 s in the Qiagen Rotorgene Q (Qiagen, Hilden). *SNORD61*, an appropriate housekeeping and endogenous reference gene, was used for the normalization of miRNA expression levels.

Ct values were exported to an excel file; the table of Ct values was uploaded to the data analysis web tool at http://www.qiagen.com/geneglobe. Fold-change is the normalized miRNA expression in each test sample divided by the normalized miRNA expression in the control sample. Patient and control groups were selected and the fold changes (2^−*ΔΔ*Ct^) of miRNAs were calculated by the online tool with ΔΔCt method (ΔCt = (Ct miRNA − Ct SNORD61), ΔΔCt = ΔCt (patient)–ΔCt (control)). Fold change values > 1 were considered to be upregulated, and fold-change values < 1 were considered to be downregulated.

### 2.3. Statistical Analysis

Statistical power analysis was performed using statistical power and sample size calculator (GPower 3.1). Gene Globe Data Analysis Center (Qiagen) was used to analyze RT-PCR data. The *P* values are calculated based on Student's *t*-test of the replicate 2^−*ΔΔ*Ct^ values for each gene in the control group and patient groups, and the *P* values less than 0.05 are indicated as significant. For the geNorm normalization method, the *P* values are calculated based on Student's *t*-test of the normalized replicate miRNA expression values for each miRNA in the control group and patient groups.

## 3. Results

### 3.1. Distinct miRNA Expression Profile in Patients with FMF Compared to Healthy Subjects

**Figure 1:**
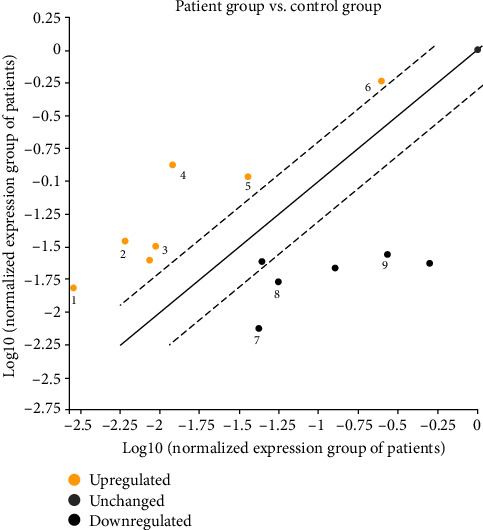
Scatter plot of significantly differentiated miRNA expressions in the patients with FMF as compared to control subjects (1: miR-340-5p, 2: miR-34a-5p, 3: miR-142-3p, 4: miR-216-5p, 5: miR-582-5p, 6: miR-429, 7: miR-569, 8: miR-1304-5p, and 9: miR-107).

**Figure 2:**
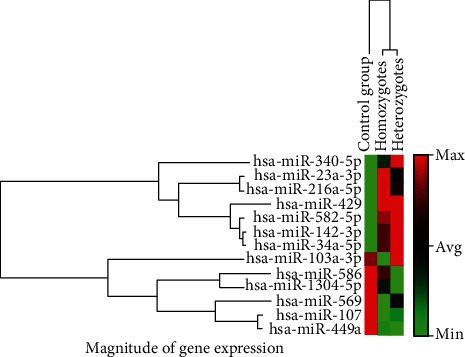
The clustergram of differential miRNAs with fold change.

The expression levels of six different miRNAs, including miR-34a-5p, miR-142-3p, miR-216a-5p, miR-340-5p, miR-429, and miR-582-5p, were found to be significantly upregulated, and three miRNAs including miR-107, miR-569, and miR-1304-5p were found to be significantly downregulated in the patient group as compared to control group (*P* value < 0.05). However, nonsignificant (*P* > 0.05) alterations of miR-23a-3p, miR-103a-3p, miR-449a, and miR-586 were determined in patients in comparison with healthy subjects (Figures 1 and 2), (Table 3). The power analysis was performed, and the power (1-*β*) turned out to be 0.83 at the effect size of 0.5 and the presented sample size.

### 3.2. Subgroup Analysis Revealed Disparate miRNA Expression in FMF Patients

**Figure 3:**
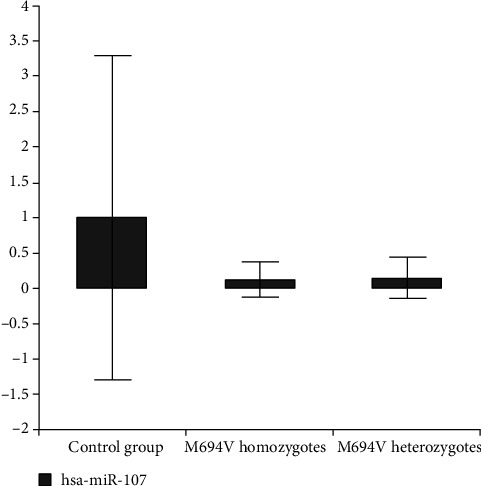
Fold change in expression of miR-107 in M694V homozygotes and heterozygotes in comparison with controls.

**Figure 4:**
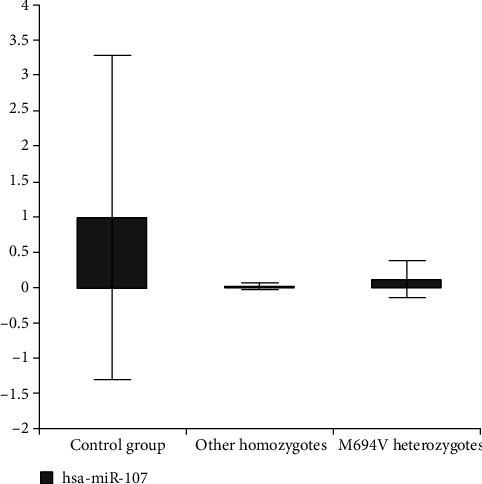
Fold change in expression of miR-107 in other homozygotes and M694V homozygotes in comparison with controls.

We created 2 subgroups; the first one consisted of patients with the homozygous genotype (M694V homozygotes and other variant's homozygotes). The second subgroup consisted of the patients with the M694V variant (homozygotes and heterozygotes). We compared the M694V homozygotes and other variant's homozygotes in the first subgroup. According to subgroup analysis, downregulation in miR-107 was more specifically in M694V homozygous mutant individuals than other homozygotes as compared to the control group (*P* = 0.03) (Figure 3) (Table 4). We also compared the M694V homozygotes and the M694V heterozygotes in the second subgroup. It was observed that the downregulation of miR-107 in M694V homozygous mutant individuals was more remarkable than that in M694V heterozygous patients compared to the control group (*P* = 0.03) (Figure 4). Again, miR-142-3p and miR-216a-5p were upregulated in the M694V homozygous mutant subgroup compared to the control group (*P* value < 0.05) than M694V heterozygous patients (Table 5).

## 4. Discussion

miRNAs are associated with the mechanisms that provide gene expression control from proinflammatory to anti-inflammatory processes in immune pathways. During the inflammatory process, various proinflammatory cytokines are expressed. Simultaneously, a large number of miRNAs are also expressed. These miRNAs act as inhibitors for multiple cytokines and activators for others. Therefore, if regulatory miRNA expression is impaired, it can lead to immunodeficiency, autoimmunity, or chronic infection [12]. In this context, FMF-miRNA association has been on the agenda in recent years, but there are limited studies. Recent findings also propounded that plasma and serum might exhibit different miRNA expression patterns in various pathological conditions. On that account, the selection of different starting material, including serum and plasma, was reported to be incomparable [17]. In this context, plasma was considered more advantageous than serum due to reproducibility and validation of the obtained results. Moreover, more miRNAs were determined in plasma samples than in serum samples [18]. Hence, we examined the plasma expression levels of miRNAs that were not previously established relations (except for miR-107 and miR-23a-3p) to FMF. In our study, miR-34a-5p, miR-142-3p, miR-216a-5p, miR-340-5p, miR-429, and miR-582-5p were upregulated and miR-107, miR-569, and miR-1304-5p were downregulated significantly (*P* < 0.05) in the FMF patients. Similar to our results, significant alterations in different miRNAs were previously reported in FMF patients. Indeed, miR-4520a was found to be strongly associated with FMF among investigated 29 different miRNAs. Moreover, the elevations in expression levels of miR-4520a were more prominent in FMF patients with M694V mutation than in healthy controls [19]. In another study, miR-204-3p expression was found as highly downregulated during the attack of FMF patients. In this study, miR-204-3p precursor molecules were transfected into macrophage-like cells that differentiated from THP-1 cells, and it was determined that various inflammasome-related pathways, including the TLR, IL-1, and IL-6 signaling pathways, were downregulated by miR-204-3p. TLRs are crucial elements of innate immunity and, when activated, lead to the transcription of genes associated with the immune and inflammatory response. Signaling with the lipopolysaccharide receptor, TLR4, differentiated various miRNAs [11]. Thus, it was suggested that miR-204-3p might have an inhibitory effect on inflammatory cytokines. Besides, *PIC3CG* which have important roles in inflammatory processes, such as proliferation, activation, and migration of inflammatory cells, was determined to be the target gene of miR-204-3p. It was also speculated that *PIC3CG* inhibition might be a target for FMF treatment by regulating TLR4-associated inflammatory cytokines (IL-12p40 and IL-6) [20]. The miRNA microarray profile was assessed on 24 patients with FMF that subgrouped due to the presence of exon 10 mutations. It has been reported that the expression patterns of miRNAs were different between FMF subgroups [8]. Again, it was reported that 14 miRNAs were expressed differently in 12 FMF patients and miR-574-3p, miR-20a-5p, let-7d-3p, and miR-197-3p had effects on inflammatory pathways. In the homozygous M694V mutation, the miR-20a-5p level was upregulated and the miR-197-3p level was downregulated and correlated with the phenotype [21].

The 15 different miRNAs associated with autoinflammatory diseases and immune response were evaluated in 51 patients with FMF, and reported miR-125a, miR-15a, miR-132, miR-155, miR-146a, miR-26a, miR-16, miR-223, miR-181a, miR-21, and miR-34a were downregulated in patients compared to the healthy subjects. In patients receiving colchicine, miR-34a, miR-146a, miR-16, miR-15a, and miR-26a levels were significantly decreased. Besides, different levels of miRNAs were observed in relapse patients. Thereby, it was observed that miRNAs might play a role in the pathogenesis of FMF via affecting the inflammatory process [22]. According to these previous findings, the present results indicated the alteration in levels of miR-34a in patients with untreated FMF patients. Interestingly, the 5p and 3p strands of miR-34 differently affected cell proliferation, invasion, and migration in cancerous cells [23]. The present data indicated the association of miR-34a-5p levels with FMF for the first time and suggested that the inhibition of miR-34a-5p could be a novel treatment strategy towards FMF. Actual data pointed out that the inhibition of miR-34a-5p might alleviate lipopolysaccharide- (LPS-) induced proinflammatory damages in human vascular endothelial cells via activation of the nuclear factor erythroid-2 related factor 2/heme oxygenase-1 (Nrf2/HO-1) pathway [24]. Nrf2 was found to play a critical role in regulating the HO-1 axis, which is known as a potent anti-inflammatory target [25]. Our findings firstly revealed the increases of miR-142-3p, miR-216a-5p, miR-340-5p, miR-429, and miR-582-5p expressions in FMF patients. These alterations were not reported in FMF cases before, and their aberrant expressions might be substantial indications of FMF pathologies related to immune responses, particularly proinflammatory factors [26–30].

A recent report revealed that 26 of 33 miRNAs associated with apoptosis including miR-15a-5p, miR-181a-5p, miR-29b-3p, miR-181b-5p, miR-214-3p, miR-365-3p, and 181c-5p were upregulated while the remaining ones (miR let-7a-5p, let-7c, let-7g-5p, miR-17b-5p, miR-15-5p, miR-16-5p, miR-23a-3p, miR-25-3p, miR-24-3p, miR-26b-5p, miR-26a-5p, miR-27a-3p, miR-30a-5p, miR-29c-3p, miR-30d-5p, miR 30e-5p, miR-195-5p, miR-106b-5p, and miR-146a-5p) were downregulated in patients with FMF. Thus, it was suggested that various miRNAs involving miR-23a-3p, which is one of our evaluated miRNAs, could play a crucial role in the pathogenesis of FMF via affecting the apoptosis-related pathways [4]. In contrast to this study, miR-23a-3p was upregulated in our patient group but it was not statistically different from the controls (*P* > 0.05).

Plasma miRNA profiling of FMF patients with homozygous M694V mutation revealed that the expression levels of miR-144-3p, miR-4454, miR-451a, and miR-21-5p were upregulated while let-7d-5p, miR-148b-3p, and miR-107 were downregulated in the FMF patients. In particular, miR-107, miR-21, and miR-148 were associated with innate immunity [31]. Inconsistent with this observation, we observed remarkable (*P* value < 0.05) downregulations in miR-107, miR-569, and miR-1304 in the FMF patients. According to the subgroup analysis, downregulation in miR-107 was significant in M694V homozygous mutant individuals compared to the control group than other homozygotes. Besides, miR-107 was downregulated in M694V homozygous mutant individuals compared to the control group than M694V heterozygous patients. miR-216a-5p and miR-142-3p were significantly upregulated in the M694V homozygous mutant subgroup compared to the control group (*P* value < 0.05) M694V heterozygous patients. Toll-like receptors (TLRs) induce several inflammatory pathways, and various miRNAs participate in regulating these pathways. It was demonstrated that signaling with the LPS receptor, TLR4, reduced the expression of miR-107 in murine macrophages [32]. However, the exact roles of miR-569 and miR-1304 were still unclear in the pathogenesis of FMF. But the expressions of miR-569 and miR-1304 were found to be consistently decreased in cancerous cells and targeted to FOS and HO-1 [33, 34]. Herewith, we speculate that the roles of downregulated three miRNAs, notably mir-107, in the inflammation process might contribute to the FMF pathogenesis.

miRNAs are regulators of the gene expression and dysregulation of the miRNAs and lead to several pathological processes. In this context, we evaluated the expressions of 13 different miRNAs. The present findings clearly propounded that miR-34a-5p, miR-142-3p, miR-216a-5p, miR-340-5p, miR-429, and miR-582-5p were upregulated while miR-107, miR-569, and miR-1304-5p were downregulated in patients with FMF. Notably, the alterations in the miR-107 level were significant in M694V homozygous mutant individuals compared to other homozygous mutants and M694V heterozygous patients. Hence, we suggested that downregulation of miR-107 might be closely associated with the M694V genotype. However, the current study has some limitations. One of the limitations of this study had to do with the relatively small number of patients. In this regard, reaching a larger number of patients, particularly for subgroups, would have enabled us to achieve more meaningful results. And also selecting more miRNAs or even making a miRNome (containing all miRNAs) would have been much more informative.

## 5. Conclusion

The present study executed the association between miRNA and FMF pathogenesis. In addition, it was concluded that mir-107 might be associated with the M694V genotype and during clinical follow-up of the patients with M694V mutation which is closely related to amyloidosis. Thus, the evaluation of mir-107 expression might be suggestive. Our findings may provide evidence for a potential therapeutic target as well as a novel diagnostic biomarker for FMF therapy. Further studies may provide novel and effective diagnostic and therapeutic agents that target examined miRNAs. Targeting miRNAs in FMF seems to be promising and may yield a new generation of rational therapeutics and diagnostic or monitoring tools enabling FMF treatment.

## Figures and Tables

**Table 1:** Demographics and characteristics feature of the FMF patients.

	Male	Female
Sex	10 (36%)	18 (64%)
Mean age	23.39 ± 14.68	
Symptoms, *n* (%)		
Abdominal pain	13 (46.4%)	
Fever	3 (10.7%)	
Arthritis/arthralgia	4 (14.3%)	
All of the symptoms	8 (28.6%)	

**Table 2:** Distribution of the mutations in the FMF patients.

Mutation	Genotype	*n* (%)
Homozygote genotypes	M694V/M694V	10 (35.7)
	E148Q/E148Q	3 (10.7)
	M680I/M680I	3 (10.7)
	P369S/P369S	1 (3.6)
Compound heterozygote genotypes	M694V/E148Q	4 (14.3)
	M680I/V726A	4 (14.3)
	M694V/P369S	1 (3.6)
	M694V/V726A	1 (3.6)
	E148Q/M680I	1 (3.6)

**Table 3:** The differential expression profiles of all examined miRNAs.

miR-ID	Average Ct				
	Control group	Patient group	Fold change	95% CI	*P* value
Snord61	20.89	22.65	1	(1.00, 1.00)	0.77
hsa-miR-107	22.77	27.84	0.10	(0.02, 0.18)	0.00^∗^
hsa-miR-569	25.46	29.71	0.18	(0.05, 0.31)	0.03^∗^
hsa-miR-340-5p	29.35	28.68	5.39	(2.27, 8.51)	0.00^∗^
hsa-miR-582-5p	25.68	25.88	2.96	(1.33, 4.59)	0.01^∗^
hsa-miR-23a-3p	27.76	27.99	2.89	(0.66, 5.13)	0.33
hsa-miR-216a-5p	27.27	25.57	11.01	(1.74, 20.28)	0.00^∗^
hsa-miR-429	22.90	23.45	2.32	(1.31, 3.34)	0.00^∗^
hsa-miR-142-3p	27.63	27.64	3.39	(1.26, 5.50)	0.04^∗^
hsa-miR-586	23.86	28.19	0.17	(0.01, 0.33)	0.21
hsa-miR-1304-5p	25.06	28.54	0.30	(0.09, 0.52)	0.03^∗^
hsa-miR-449a	21.90	28.06	0.05	(0.00, 0.11)	0.08
hsa-miR-34a-5p	28.26	27.50	5.73	(1.97, 9.49)	0.00^∗^
hsa-miR-103a-3p	25.39	28.03	0.55	(0.12, 0.97)	0.13

^∗^Significant statistical difference of miRNA expression between the patient and control groups (*P* < 0.05).

**Table 4:** Downregulation of miR-107 expression due to M694V homozygosity.

	Average Ct ± standard deviation (fold change)		
	Control group	Other variant's homozygotes	M694V homozygotes
Snord61	20.89 ± 1.04	22.81 ± 0.48	22.31 ± 0.77
hsa-miR-107	22.77 ± 2.78	30.14 ± 1.57 (0.02)	27.18 ± 1.96^∗^ (0.13)

^∗^Significant statistical difference of miRNA expression between M694V homozygotes and other variant's homozygotes (*P* < 0.05).

**Table 5:** Alterations in miR-107, mir-216a-5p, and miR-142-3p expressions due to homozygosity and heterozygosity of M694V.

	Average Ct ± standard deviation (fold change)		
	Control group	M694V homozygotes	M694V heterozygotes
Snord61	20.89 ± 1.04	22.31 ± 0.77	22.64 ± 0.61
hsa-miR-107	22.77 ± 2.78	27.18 ± 1.96^∗^ (0.13)	27.29 ± 1.43 (0.15)
hsa-miR-216a-5p	27.27 ± 1.94	25.51 ± 2.47^∗^ (9.08)	26.45 ± 0.69 (5.95)
hsa-miR-142-3p	27.63 ± 1.44	27.50 ± 1.74^∗^ (2.95)	27.76 ± 0.71 (3.07)

^∗^Significant statistical difference of miRNA expression between M694V homozygotes and heterozygotes (*P* < 0.05).

## Data Availability

The data is available upon request to Dr. Cigdem Yuce Kahraman (cigdem.kahraman@atauni.edu.tr) through a data access committee, institutional review board, or the authors themselves.
